# Adrenocortical control in the neonatal rat: ACTH- and cAMP-independent corticosterone production during hypoxia

**DOI:** 10.1002/phy2.54

**Published:** 2013-08-22

**Authors:** Karl Johnson, Eric D Bruder, Hershel Raff

**Affiliations:** 1 Endocrine Research Laboratory, Aurora Research Institute, Aurora St. Luke's Medical CenterMilwaukee, Wisconsin; 2 Department of Medicine, Surgery, and Physiology, Medical College of WisconsinMilwaukee, Wisconsin

**Keywords:** ACTH, adrenal cortex, cAMP, cGMP, corticosteroid-binding globulin, hypothalamic–pituitary–adrenal axis, newborn, oxygen

## Abstract

We have previously demonstrated that the neonatal corticosterone response to acute hypoxia shifts from ACTH independence to ACTH dependence between postnatal days two (PD2) and eight (PD8). Cyclic AMP (cAMP) is the obligatory intracellular second messenger of ACTH action, and we hypothesized that corticosterone production in neonatal rats shifts from a cAMP-independent mechanism to cAMP-dependent mechanism between PD2 and PD8. Plasma ACTH and corticosterone and adrenal cAMP and cGMP responses to acute severe hypoxia (8% O_2_ for 5, 10, 20, 30, and 180 min) were measured in neonatal rats at PD2, PD8, and PD15. Plasma ACTH and corticosterone were measured by radioimmunoassay, and adrenal cAMP and cGMP were measured by ELISA. Plasma corticosterone-binding globulin (CBG) was measured in normoxic pups by ELISA. The largest corticosterone response was observed in PD2 pups, despite only a small increase in plasma ACTH that was not sustained. The PD2 ACTH-independent increase in corticosterone occurred with no change in adrenal cAMP or cGMP content. Plasma CBG concentration was lowest in PD2 pups. Large corticosterone responses were measured during the first 30 min of hypoxia. Differences in corticosterone responses between PD2 and PD8 pups cannot be attributed to changes in plasma protein binding capacity, and the PD2 corticosterone response is consistent with a nongenomic mechanism of action. We conclude that the sustained corticosterone response to hypoxia in PD2 pups occurs with small and transient ACTH responses and independently of increases in adrenal cAMP or cGMP.

## Introduction

Acute hypoxia is a frequent complication in neonates, primarily as a result of prematurity, cardiopulmonary disease, or respiratory infection (Frankel and Stevenson 1987; Low et al. 1993). The clinical consequences of acute hypoxia are severe, and medical interventions such as ventilator support, oxygen therapy, and corticosteroid use are often necessary to prevent acute and long-term hypoxic injury (Tin 2004; Nye 2007; Tin and Wiswell 2008). The number of premature births in the U.S. has increased by 30% in the last two decades, making it increasingly important to better understand the integrated neonatal response to acute hypoxia (Martin et al. 2007).

It is known that the neonatal response to acute hypoxia involves the hypothalamic–pituitary–adrenal (HPA) axis, resulting in an increase in plasma glucocorticoids that promotes lung maturation and improves the vasoconstrictor response to circulating catecholamines (Morales et al. 1998; Watterberg et al. 1999; Deruelle et al. 2003). The rat adrenal zona fasciculata/reticularis produces the glucocorticoid corticosterone through a well-documented ACTH-dependent cAMP-stimulated pathway (Gallo-Payet and Payet 2003). While a number of other minor mediators of corticosterone production, such as calcium, have been identified, cAMP remains the established main second messenger responsible for corticosterone production (Gallo-Payet and Payet 2003).

We have previously demonstrated that the neonatal corticosterone response to acute hypoxia shifts from ACTH independence to ACTH dependence between postnatal days two (PD2) and eight (PD8) (Bruder et al. 2008). The mediator(s) of the ACTH-independent increase in corticosterone in response to hypoxia in the newborn have yet to be identified. In concert with this shift from ACTH independence to dependence, we hypothesize that the neonatal cellular adrenocortical cAMP response to acute hypoxia follows the same pattern between PD2 and PD8, and that measurement of adrenal cAMP will give insight into the potential mechanisms and secretagogues involved in this novel activation of adrenocortical function. Furthermore, the measurement of responses during the first 30 min of hypoxia will be helpful in ruling out transcriptional mechanisms involved in adrenal control.

The current study evaluated plasma ACTH, and corticosterone concentrations and adrenal cAMP and cGMP content at 0, 30, and 180 min of hypoxia in PD2 and PD8 rat pups. Additional experiments evaluated plasma ACTH and corticosterone at 5, 10, 20, and 30 min of hypoxia. Plasma corticosteroid-binding globulin (CBG) concentrations in PD2, 8, and 15 pups were also evaluated to rule out changes in plasma steroid binding as an explanation of ACTH-independent increases in plasma corticosterone. Pulse oximetry measurements were obtained to assess the cardiopulmonary response to acute hypoxia.

## Methods

### Animal treatment and experimental protocol

The Aurora Health Care Institutional Animal Care and Use Committee approved the animal protocol. Timed-pregnant Sprague-Dawley rats at gestational day 15 (*n* = 42) were obtained from Harlan Sprague Dawley (Indianapolis, IN) and were maintained on a standard diet and water ad libitum in a controlled environment (0600–1800 lights on). Pups were studied on the morning of postnatal days 2, 8, and 15 (PD2, PD8, and PD15). Pups were separated from their dam immediately prior to experimentation and randomly divided into two equal groups for normoxia (control) or hypoxia (12–13 pups/group; mixed sexes). The pups were placed in environmental chambers (21% O_2_; room air) for 10 min prior to the start of the hypoxic period. Chamber O_2_ concentration was monitored with TED-60T O_2_ sensors (Teledyne Analytical Instruments, City of Industry, CA). Pups were placed on adequate bedding and allowed free range of motion and room to huddle throughout the experiment. A variable control heating pad (Moore Medical, Farmington, CT) was placed beneath the bedding and kept at the lowest setting required to maintain body temperature in normoxic controls (Guenther et al. 2012). One sentinel pup per group was instrumented to monitor body temperature using RET-3-Iso rectal probes and a BAT-12 digital thermometer connected to a SBT-5 switchbox (Physitemp Instruments, Clifton, NJ). The same sentinel pups were fixed with a collar sensor and transcutaneous O_2_ saturation (SpO_2_) was monitored using the MouseOx Plus Small Animal Vital Signs Monitor (Starr Life Sciences, Oakmont, PA). Sentinel pups were not used for plasma or tissue analysis.

### Sample collection

Baseline measurements were obtained at the end of the 10-min prehypoxia period, at which time some pups were removed from the chamber and sacrificed. Trunk blood from 2–3 pups was pooled in one EDTA-plasma tube and treated as one sample. Adrenal glands were removed, flash frozen in liquid nitrogen, and stored at −70°C until later analysis. Immediately following the 10 min prehypoxia period, the input oxygen concentration was decreased to 8% (room air + N_2_; 8 L/min). Depending on experiment, pups were removed from the chamber at 5, 10, 20, 30, or 180 min of hypoxia and decapitated. Pups used for plasma CBG analysis were exposed to room air (normoxia) for 180 min and then decapitated.

### Plasma hormone, adrenal second messenger, and plasma CBG assays

Plasma ACTH and corticosterone were measured by radioimmunoassay (MP Biomedicals, Orangeburg, NJ) as described previously (Raff et al. 2003a). Frozen adrenal tissue samples were pulverized using a liquid nitrogen-cooled mini mortar (Bel-Art Products, Pequannock, NJ) and immediately used for second messenger content analysis. Adrenal cAMP and cGMP content were determined by ELISA according to the manufacturer's protocol (Arbor Assays, Ann Arbor, MI). PD15 cAMP and cGMP extracts were diluted 1:10 with provided assay sample diluent. Optical densities of both assays were measured at 450 nm. Adrenal content of cAMP and cGMP were calculated by interpolating against a known standard curve ranging from 0.617 to 150 pmol/mL and 0.5 to 32 pmol/mL, respectively. Cyclic AMP intra- and interassay precision was 8.6–12.3% and 10.0–11.3%, respectively, and cGMP intra- and interassay precision was 6.3–8.6% and 6.5–8.3%, respectively. Adrenal cAMP and cGMP data are expressed as pmol per adrenal gland; we did not want to take the time to weigh the adrenal glands to ensure immediate freezing to retain cyclic nucleotide concentration. Plasma CBG was measured by ELISA according to the manufacturer's protocol (USCN Life Science Inc., Houston, TX). Samples were diluted 1:1000 in PBS (Quality Biological Inc., Gaithersburg, MD). The concentration of CBG was calculated by interpolating against a known standard curve ranging from 3.12 to 200 ng/mL.

### Statistical analysis

Hormone, second messenger, and CBG data were analyzed by two-way ANOVA. SpO_2_ data were analyzed by two-way ANOVA for repeated measures. All post hoc analyses were performed by Student–Newman–Keuls method for multiple comparisons (Sigma Stat 2.03, Systat, San Jose, CA).

## Results

Baseline SpO_2_ was 96.3 ± 1.9% (*N* = 6) in PD2 and 98.8 ± 0.5% (*N* = 5) in PD8 pups. Both PD2 and PD8 pups experienced significant oxygen desaturation, with PD2 rat pups experiencing a greater decrease in oxygen saturation at 20 min than PD8 (49.0 ± 6.3% vs. 67.4 ± 3.1%, respectively; *P* = 0.004).

**Figure 1:**
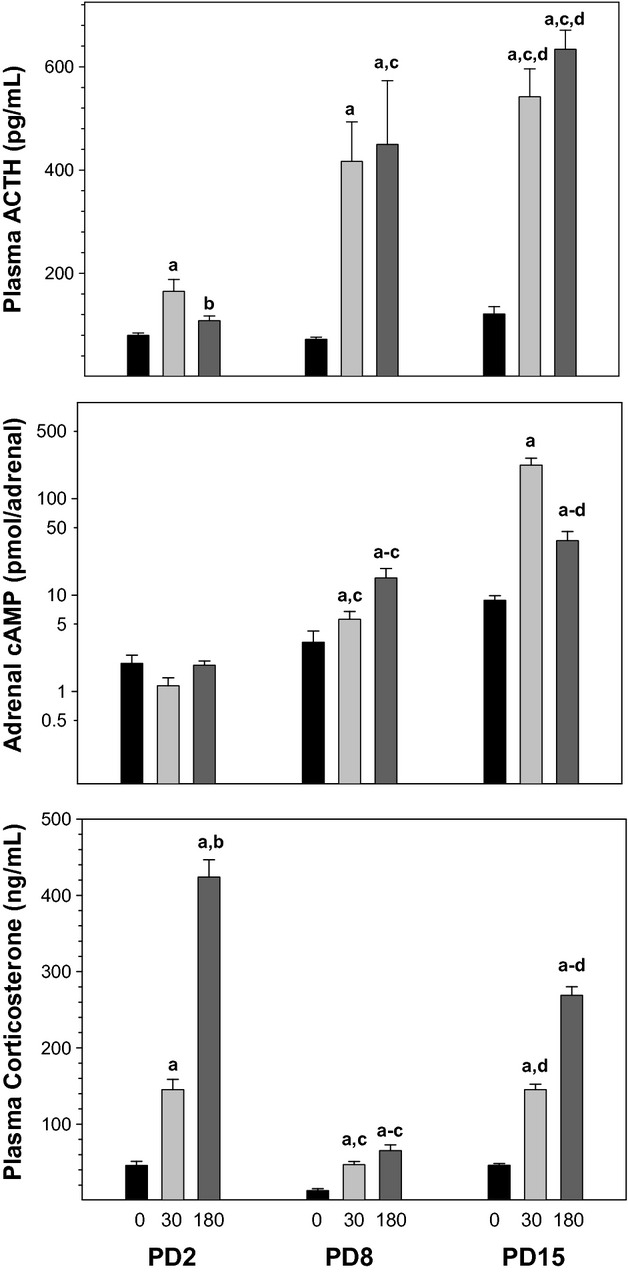
Plasma ACTH, adrenal cAMP, and plasma corticosterone responses to hypoxia. Postnatal day (PD) 2, PD8, and PD15 rat pups were exposed to 8% O_2_ for a maximum of 180 min. Blood and tissue collection was performed at 0, 30, and 180 min. ^a^Different from 0 min within age (*P* < 0.05); ^b^different from 30 min within age (*P* < 0.05); ^c^different from PD2 within time point (*P* < 0.05); ^d^different from PD8 within time point (*P* < 0.05). *N* = 6–10 measurements per time point for each age studied.

PD2 pups had a small but significant ACTH response to hypoxia at 30 min (*P* < 0.001), but not at 180 min (Fig. 1). PD8 pups at 180 min and PD15 pups at 30 (*P* < 0.001) and 180 min (*P* < 0.001) had significantly larger ACTH responses to hypoxia compared with PD2 pups. Furthermore, PD15 pups had significantly larger ACTH responses than PD8 pups at both 30 (*P* = 0.009) and 180 min (*P* = 0.038). These data show an overall pattern of progressively larger ACTH responses to hypoxia as age and time increases, with a very small but significant ACTH response in PD2 pups that was not sustained over the 180 min experimental period.

Adrenal cAMP content did not increase in response to hypoxia in PD2 pups (Fig. 1). This was different from the other age groups, as both PD8 and PD15 pups had significant increases in cAMP content in response to hypoxia. Again, the overall trend of increasing cAMP response to hypoxia with age and time was observed with PD2 pups showing no cAMP response to hypoxia. After normalizing cAMP content assuming age-appropriate adrenal weight, basal cAMP was not different between age groups when expressed as pmol/mg of adrenal weight.

Despite the above findings in PD2 pups (a small ACTH response and no adrenal cAMP response to hypoxia), plasma corticosterone concentrations in PD2 pups were significantly increased at 30 (*P* < 0.001) and 180 (*P* < 0.001) min of hypoxia (Fig. 1). The corticosterone response at 30 min of hypoxia was greater in PD2 pups compared with PD8 (*P* < 0.001) and similar to PD15. At 180 min of hypoxia, PD2 plasma corticosterone levels were also higher than PD8 (*P* < 0.001) and PD15 (*P* < 0.001).

**Figure 2:**
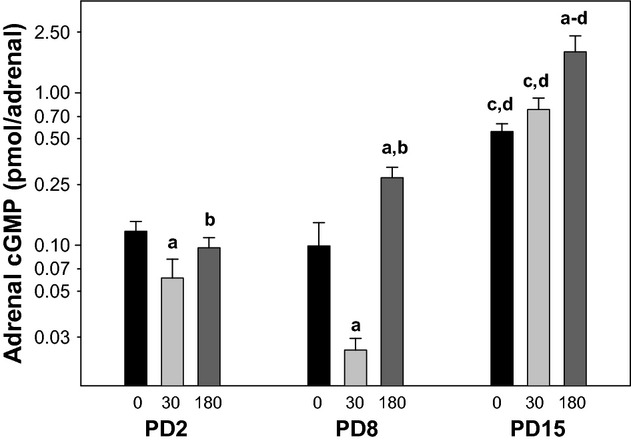
Adrenal cGMP response to hypoxia. Postnatal day (PD) 2, PD8, and PD15 rats were exposed to 8% O_2_ for a maximum of 180 min. Blood and tissue collection was performed at 0, 30, and 180 min. ^a^Different from 0 min within age (*P* < 0.05); ^b^different from 30 min within age (*P* < 0.05); ^c^different from PD2 within time point (*P* < 0.05); ^d^different from PD8 within time point (*P* < 0.05). *N* = 6–10 measurements per time point for each age studied.

Adrenal cGMP content data for PD2, 8, and 15 pups exposed to 8% O_2_ for 0, 30, and 180 min are shown in Figure 2. There was no increase in adrenal cGMP content in PD2 pups in response to hypoxia. In PD8 pups, cGMP was initially decreased at 30 min (*P* = 0.017) but, by 180 min of hypoxia, a significant increase was observed (*P* < 0.001). Adrenal cGMP content was higher in PD15 compared with younger age groups. This difference was not observed when cGMP was normalized to estimated adrenal weight (pmol/mg of adrenal weight). There was no change in cGMP content in response to hypoxia in PD15 pups.

**Figure 3:**
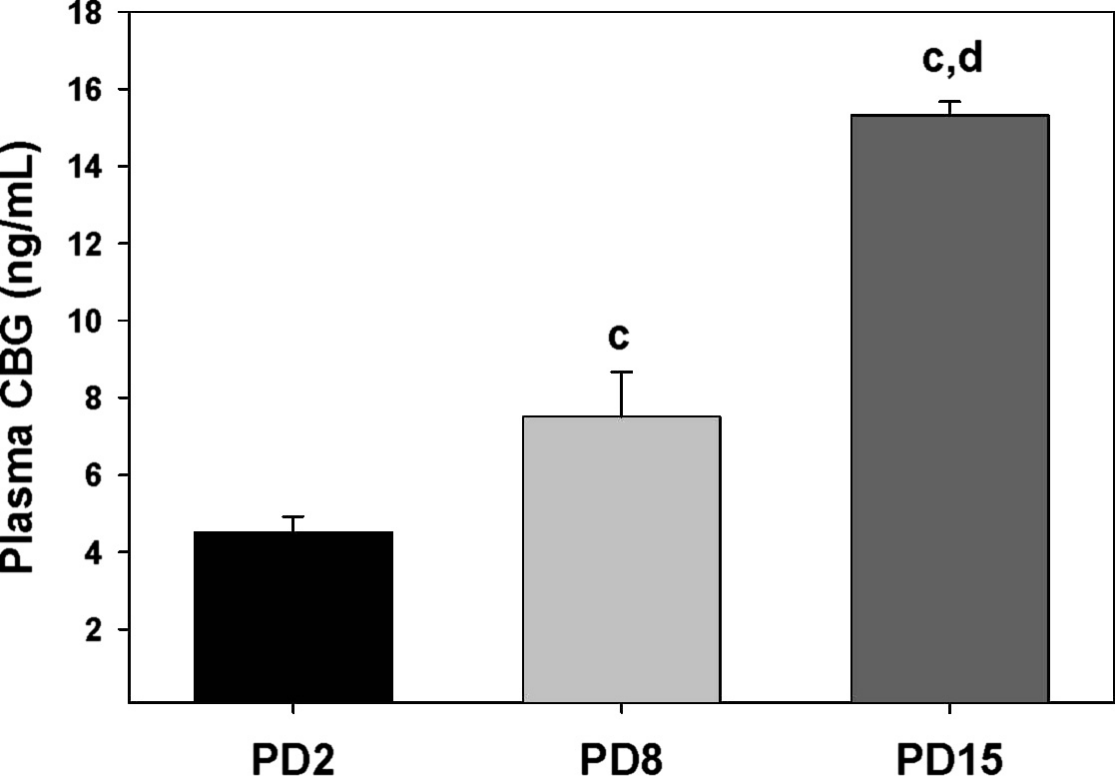
Plasma CBG. Postnatal day (PD) 2, PD8, and PD15 rats were exposed to room air for 180 min (time control). ^c^Different from PD2 within time point (*P* < 0.05); ^d^different from PD8 within time point (*P* < 0.05). *N* = 7 measurements per time point for each age studied.

Plasma CBG data for PD2, PD8, and PD15 pups exposed to room air (normoxic time control) for 180 min are shown in Figure 3. Plasma CBG concentration increased with age, as the PD8 CBG concentration was higher than PD2 (*P* = 0.004), and the PD15 concentration was higher than PD8 (*P* < 0.001).

**Figure 4:**
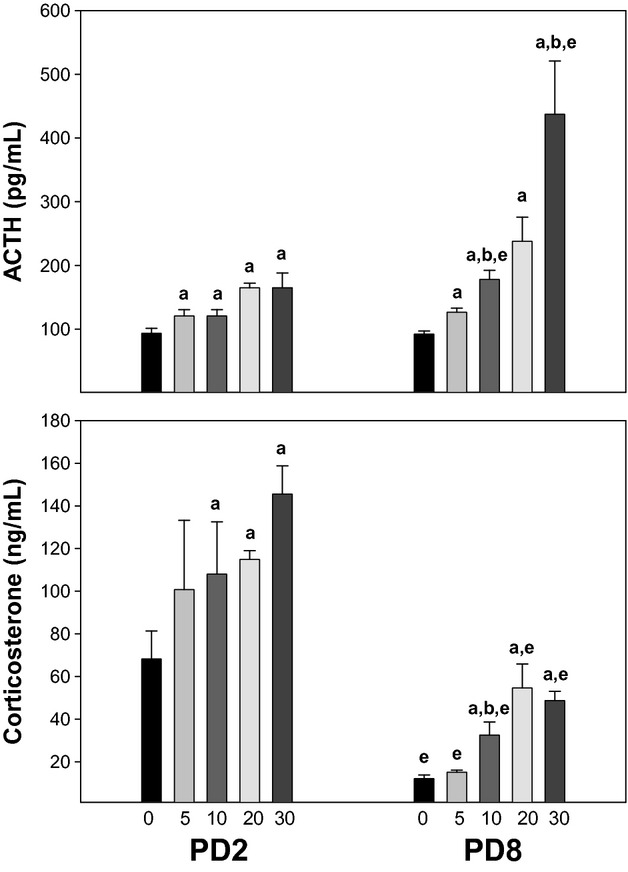
Rapid plasma ACTH and corticosterone responses to hypoxia. Postnatal day (PD) 2 and PD8 rats were exposed to 8% O_2_ for 0, 5, 10, 20, and 30 min. ^a^Different from 0 min within age; ^b^different from 5 min within age group; ^e^different from PD2 within time point. *N* = 7 for each age studied.

Plasma ACTH and corticosterone data for PD2 and PD8 pups exposed to 8% O_2_ for 5, 10, 20, and 30 min are shown in Figure 4. PD2 pups showed a small but significant increase in ACTH during these very brief exposures to hypoxia. Larger ACTH responses were observed in PD8 at 10 min (*P* = 0.027) and 30 min (*P* < 0.001) compared with PD2 pups, and progressively higher ACTH concentrations were observed in PD8 pups as the duration of hypoxia increased. Baseline corticosterone was higher in PD2 compared with PD8 pups (*P* = 0.004) and PD2 pups showed progressively larger corticosterone responses as time progressed. Similar to the findings in Figure 1, PD2 pups had significantly larger corticosterone responses to hypoxia compared to PD8 pups despite smaller plasma ACTH responses in P2D2 pups.

## Discussion

The purpose of this study was to address the hypothesis that corticosterone production in the neonatal rat shifts from a cAMP-independent mechanism to cAMP-dependent mechanism between PD2 and PD8, in parallel with our previous data showing ACTH-independent responses in the PD2 pups (Bruder et al. 2008). Our results confirm this hypothesis and reinforce the notion that the adrenocortical response to hypoxia in the early neonatal period is likely to be ACTH independent (Bruder et al. 2008). Small but significant increases in plasma ACTH concentrations were measured in PD2 pups early in the hypoxic exposure; however, when compared to PD8 and PD15 pups, the ACTH response was considerably less. Furthermore, at 180 min of hypoxia, ACTH was no longer increased from baseline in PD2 pups. The corticosterone data were also consistent with previous studies where a large response was measured in PD2 pups, despite a small response in PD8 pups (Bruder et al. 2008). Adrenal cGMP content did not significantly change in PD2 pups, and did not support cGMP as a mechanism for ACTH-independent corticosterone production. Plasma CBG concentration increased with age in our study, indicating that the larger corticosterone response to hypoxia in PD2 pups could not be accounted for by higher plasma protein binding capacity, when compared to older pups.

The neonatal rat undergoes many changes in early life that result in the maturation of HPA axis control and capacity to fully produce corticosterone (Nagaya et al. 1995; Zilz et al. 1999). We have previously described the effects of chronic and acute hypoxia on the HPA axis in the developing neonatal rat (Raff et al. 2003a,b, 2004; Bruder et al. 2008, 2011). Our primary focus has been to evaluate the change in control of adrenal corticosterone production that shifts from ACTH independence to ACTH dependence between PD2 and PD8. The ACTH-independent corticosterone response in PD2 pups may be unique to hypoxia as a stressor as adrenal cells from PD1 rats generate a corticosterone response in vitro in response to ACTH and cAMP (Arai and Widmaier 1993). In the current experiments, we chose to measure adrenal cAMP content, the main second messenger responsible for corticosterone production in response to ACTH (Gallo-Payet and Payet 2003). If cAMP increased in response to hypoxia in PD2 pups, that would imply that ACTH receptor-dependent mechanisms were in control and that, perhaps, circulating bioactive ACTH was increasing despite no increase in immunological active ACTH. A dissociation of bioactive and immunoactive ACTH has been described previously (Engeland et al. 1989). The lack of a cAMP response in the PD2 strongly suggests that the mechanism of the sustained corticosterone response to hypoxia is likely to be ACTH independent.

We have previously focused on other components of the HPA axis to explain this phenomenon. In experiments of *chronic* hypoxia (12% O_2_ for 7 days), we have shown that the resulting prolonged glucocorticoid exposure did not decrease expression or anatomic distribution of paraventricular CRH and AVP, the known hypothalamic controllers of ACTH release (Raff et al. 2003a). Again, with *chronic* hypoxia (12% O_2_ for 7 days), we have also shown that the neonatal pituitary gland is sensitive to glucocorticoid negative feedback, and, as a result of hypoxia-induced increases in corticosterone, has an attenuated ACTH response to exogenous corticotropin-releasing hormone (Raff et al. 2003a).

There are other potential non-ACTH mechanisms for controlling corticosteronogenesis. They include sympathetic input via the splanchnic nerves (Walker 1995; Engeland 1998) and/or local production of factors such as VIP and catecholamines (Bodnar et al. 1997; Bornstein et al. 2008). We have investigated whether adrenocortical sympathetic input could explain the ACTH-independent corticosterone response to chronic hypoxia from birth in PD7 rat pups; chemical sympathectomy with guanethidine did attenuate hypoxia-induced stimulation of corticosterone, while exogenous ACTH remained capable of stimulating corticosterone in PD7 pups (Raff et al. 2004). It remains possible that neural input could stimulate corticosterone in the PD2 rat.

The current studies of acute hypoxia are more relevant to clinical situations in neonatal humans, particularly in prematurity, in which bouts of acute hypoxia are relatively common, but chronic hypoxia can be minimized with clinical intervention (Stokowski 2005; Martin and Wilson 2012). Our previous studies with acute hypoxia (8% O_2_ for up to 4 hours) have measured the change in expression of adrenal StAR protein*,* the main regulatory protein of rate-limiting cholesterol transport into the corticosterone synthetic pathway (Ariyoshi et al. 1998). These experiments showed no change in *Star* mRNA expression in PD2 (Bruder et al. 2008, 2011). However, transcriptional changes in *Star* mRNA are not necessarily required for an increase in corticosterone production because of the release of labile stores of StAR protein (Ariyoshi et al. 1998), so it remains to be determined if the increase in corticosterone production during hypoxia in PD2 pups is StAR protein mediated.

Adrenal cAMP is the obvious second messenger to study, as after many decades of research on the cellular control of corticosterone production, cAMP has proven to be the obligatory and most important second messenger of ACTH action (Gallo-Payet and Payet 2003). Our results showed a small *decrease* in adrenal content of cAMP at 30 min of hypoxia, and no significant change at 180 min in PD2 pups. These results indicate that the primary cellular mechanism normally stimulated by ACTH was quiescent, yet a very large corticosterone response was measured in PD2 pups in response to hypoxia. The results also showed a significant increase in adrenal cAMP content in response to hypoxia in PD8 pups. This indicates a difference in cellular mechanisms between PD2 and PD8 rats exposed to hypoxia, and supports our hypothesis that corticosterone production shifts from a cAMP-independent mechanism to cAMP-dependent mechanism between PD2 and PD8.

It is remarkable and intriguing how the hypoxic PD2 rat can mount such a large corticosterone response without a measurable change in adrenal cAMP content. First, the melanocortin 2 (ACTH) receptor does not appear to be activated, as it is well established that the primary cellular response results in an increase in adrenal cAMP production (Gallo-Payet and Payet 2003). Second, previous explanations for the ACTH-independent corticosterone response observed in PD2 pups have included the possibility of increased sensitivity of adrenal cortical tissue to ACTH in PD2 compared with PD8 (Chintamaneni et al. 2013). Studies have demonstrated a period from PD4-PD14, termed the stress hyporesponsive period (SHRP), during which ACTH and corticosterone responses to stress are attenuated (Denenberg and Bell 1960; Walker et al. 1986; Walker and Aubert 1988; Levine et al. 1991). It was thought that the PD2 model may lie outside of this SHRP, and differences in ACTH and corticosterone response could be explained by differences in ACTH sensitivity (Chintamaneni et al. 2013). The cAMP results in PD2 pups make this possibility unlikely as increases in PD2 cAMP would be expected in this scenario. Lastly, the absence of a cAMP response makes ACTH an unlikely corticotrophic candidate in the hypoxic PD2 pup. It is more likely that a non-ACTH, non-melanocortin 2 receptor-mediated mechanism controls the corticosterone response to hypoxia in the newborn rat.

Adrenal cGMP content did not significantly change in PD2 pups. There was an increase in PD8 pups, and a notable difference between the two ages, but these results do not support cGMP as a mechanism for ACTH-independent corticosterone production in PD2 pups. ACTH can stimulate other signaling pathways such as the mitogenic activating protein kinase (MAPK), inositol phosphate, and diacylglycerol (Gallo-Payet and Payet 2003). Although these pathways have been shown to be less potent than cAMP, they are potential candidates for future studies (Gallo-Payet and Payet 2003).

PD2 pups had the largest corticosterone response to hypoxia despite the lack of a large ACTH stimulus or change in adrenal cAMP content consistent with previous studies (Bruder et al. 2008, 2011). We measured plasma CBG concentration in normoxic pups to determine if plasma corticosterone binding capacity could explain this paradox. The results showed increasing levels of CBG with age, and therefore an inverse relationship to the corticosterone response to hypoxia. A steady increase in serum CBG has been observed in human neonates from birth through PD30, even in premature infants that are similar physiologically to the altricial nature of newborn rats (Kari et al. 1996). Although we have not found prior studies of CBG concentrations in rat pups as early as PD2, it has been demonstrated that corticosterone binding in plasma is very low in PD4 pups (Henning 1978). This study is important because it eliminates non-CBG plasma mediated binding of corticosterone as the explanation for our results as it evaluated binding by isotopic corticosterone binding and charcoal stripping of plasma rather than a direct measurement of CBG by immunoassay as in our study. Therefore, plasma CBG concentration does not explain the ACTH-independent increases in plasma corticosterone in PD2 pups.

Plasma ACTH and corticosterone results from very early in the acute exposure to hypoxia (5–20 min) were measured to evaluate if adrenocortical transcriptional mechanisms could theoretically be attributed to the PD2 corticosterone response. The nuclear transcriptional factor SF-1 (steroidogenic factor 1) has been shown to be a stimulator of steroidogenesis under stress by upregulating 21-hydroxylase (P450c21) and ACTH receptor expression (Beuschlein et al. 2002). Alternatively, Dax-1 (dosage-sensitive sex reversal-adrenal hypoplasia congenita critical region on the X chromosome) has been shown to inhibit steroidogenesis under stress (Beuschlein et al. 2002). The increase in steroidogenesis we observed in response to the first 20 min of hypoxia are consistent with a non-genomic mechanism of action as an explanation for the PD2 corticosterone response.

## Conclusions

We have shown that the sustained corticosterone response to hypoxia in PD2 rat pups occurred with small, transient increases in plasma ACTH and independently of changes in adrenal cAMP content. These results support an ACTH receptor-independent control of corticosterone production in the hypoxic PD2 rat. These results add to previous findings that may explain dissociations of ACTH and corticosterone levels. The ectopic expression of hormone receptors on the adrenal gland, and the action of neuropeptides, neurotransmitters, cytokines, and adipokines on adrenal cells are all potential non-ACTH stimulants of adrenal corticosterone production (Bornstein et al. 2008). Future study of the neonatal HPA axis response to acute hypoxia is important as hypoxia is a common occurrence in neonates, and the short- and long-term health complications are severe (Zhang 2005; Raff et al. 2007).
